# Linking Genes to Traits in Fungi

**DOI:** 10.1007/s00248-021-01687-x

**Published:** 2021-01-22

**Authors:** A. L. Romero-Olivares, E. W. Morrison, A. Pringle, S. D. Frey

**Affiliations:** 1 Department of Natural Resources and the Environment, University of New Hampshire, Durham, NH 03824 USA; 2 Present Address: Department of Biology, New Mexico State University, Las Cruces, NM 88001 USA; 3 Department of Botany and Department of Bacteriology, University of Wisconsin-Madison, Madison, WI 53706 USA

**Keywords:** Decomposition, Frequencies, Fungi, Gene, Genomes, Nitrogen, Traits

## Abstract

Fungi are mediators of the nitrogen and carbon cycles in terrestrial ecosystems. Examining how nitrogen uptake and organic matter decomposition potential differs in fungi can provide insight into the underlying mechanisms driving fungal ecological processes and ecosystem functioning. In this study, we assessed the frequency of genes encoding for specific enzymes that facilitate nitrogen uptake and organic matter decomposition in 879 fungal genomes with fungal taxa grouped into trait-based categories. Our linked gene-trait data approach revealed that gene frequencies vary across and within trait-based groups and that trait-based categories differ in trait space. We present two examples of how this linked gene-trait approach can be used to address ecological questions. First, we show that this type of approach can help us better understand, and potentially predict, how fungi will respond to environmental stress. Specifically, we found that trait-based categories with high nitrogen uptake gene frequency increased in relative abundance when exposed to high soil nitrogen enrichment. Second, by comparing frequencies of nitrogen uptake and organic matter decomposition genes, we found that most ectomycorrhizal fungi in our dataset have similar gene frequencies to brown rot fungi. This demonstrates that gene-trait data approaches can shed light on potential evolutionary trajectories of life history traits in fungi. We present a framework for exploring nitrogen uptake and organic matter decomposition gene frequencies in fungal trait-based groups and provide two concise examples on how to use our framework to address ecological questions from a mechanistic perspective.

**Supplementary Information:**

The online version contains supplementary material available at 10.1007/s00248-021-01687-x.

## Introduction

Trait-based ecology merges evolution and classic population and community ecology, with a focus on traits rather than species identity. The goal is to better understand the strategies species use to exploit resources by studying their most critical attributes [1]. A trait can be any attribute that impacts an organism’s performance (e.g., gene abundance, growth rate, morphology, nutritional strategy) [2]. In microbial ecology, trait-based approaches promise to enhance our ability to understand “the adaptive mechanisms driving ecology and evolution” [3] and to provide mechanistic explanations to microbially mediated ecosystem functioning. In addition, trait-based approaches may be especially useful because they can be used to infer function at a time when the majority of fungal species have yet to be named or categorized ecologically. Here we focus on fungi which mediate nitrogen (N) cycling and organic matter (OM) decomposition in terrestrial ecosystems—two of the most important processes needed to sustain ecosystem functions [4, 5]. Nitrogen uptake and OM decomposition are ubiquitous traits in fungi but the extent to which these processes are carried out varies across taxa. For example, all fungi require exogenous N for growth and survival, but not all can utilize all forms of N [6]. Similarly, many fungi can metabolize OM but only some taxa have the capacity to decompose complex organic compounds such as lignin [7]. The differential distribution of these traits can influence the functioning of the ecosystems in which they reside. Indeed, in fungal ecology, trait-based approaches have enriched our understanding of trait variation among fungi and how these traits influence their ecology. For instance, growth rate varied broadly across 1582 fungal isolates, but regardless of this variation, this trait was a strong predictor of wood decomposition [8].

FUNGuild is a tool that links fungal taxonomical molecular identity (i.e., ITS) to ecological function by grouping fungal taxa into four trait-based categories: (1) trophic mode, where fungi are grouped as symbiotrophs*,* saprotrophs, or pathotrophs; (2) functional guild based on the class of environmental resources exploited: animal pathogens, ectomycorrhizal (ECM) fungi, endophytes, plant pathogens, undefined saprotrophs (i.e., general saprotrophs), and wood saprotrophs; (3) growth morphology, where fungi are classified according to their morphological phenotype, such as agaricoids, boletoids, corticioids, gasteroids, microfungi, polyporoids, and yeasts (including facultative yeasts); and (4) rot type based on the ability to decompose certain types of organic compounds, namely, brown rot, soft rot, and white rot fungi [9]. This tool has been widely used to explore ecological patterns in high-throughput sequencing data of fungal communities. For example, climate was observed to be an important driver of the biogeography of trait-based fungal groups, with mycorrhizal fungi appearing to have a narrower climatic tolerance in contrast to pathogenic fungi which tolerate broader climatic conditions [10]. Growth morphology was shown to be a key factor influencing the biogeography of fungal communities and how fungi respond to abiotic conditions, irrespective of taxonomic identity [11].

While FUNGuild groups fungal taxa by their functional attributes, MycoCosm is a fungal genomic database that provides access to the complete genomes of over 1000 fungal taxa [12]. Treseder and Lennon [13] used this database to identify key relationships among genes involved in stress tolerance, N uptake, and OM decomposition and linked these relationships to fungal growth morphology. More recently, Zanne and collaborators [14] expanded on this, describing how trait variation and gene copy number of key proteins involved in OM decomposition and stress tolerance vary across functional guilds of plant-associated fungi. Similarly, Nagy et al. [15] identified six fungal traits and linked them to genes and gene families associated with the development of fruiting bodies, mycorrhizal life strategy, and the ability to decompose wood, among others. However, applying this knowledge to advance ecological research has remained a challenge.

In this study, we aimed to showcase how linking genes to traits in fungi can be used to forward ecological research. We assessed the frequency of genes encoding for specific enzymes that facilitate N uptake and OM decomposition in nearly 1000 fungal genomes obtained from MycoCosm, with fungal taxa grouped into FUNGuild trait-based categories. We then examined how these gene frequencies compared and contrasted among fungal trait-based categories. We present two examples showing how this linked gene-trait data can be used to address questions in ecological research. In the first example, we used previously published fungal RNA data collected from the Chronic Nitrogen Amendment Study (CNAS) at the Harvard Forest Long-term Ecological Research (LTER) site, in conjunction with the gene frequency data obtained from MycoCosm, to address the question: does knowing something about the frequencies of N uptake and OM decomposition genes of fungal functional groups provide insight into expected fungal responses under simulated N fertilization? This question was motivated by decades of research showing that in temperate forest ecosystems, simulated N deposition often results in OM accumulation [16–18] due to a combination of mechanisms [19–22], including fungal community shifts [23].

In the second example, we examined the frequencies of N uptake and OM decomposition genes in 62 different ectomycorrhizal (ECM) fungal taxa to examine how gene frequencies vary within a single functional guild and to ask the question: what can we learn about ECM life history traits by comparing the frequencies of N uptake and OM decomposition genes to those of fungi categorized by rot type (i.e., brown, soft, or white rot)? This question derived from evidence showing that the evolutionary history of ECM fungi is polyphyletic [24]. That is, ECM fungi are symbiotrophs that evolved multiple times from ~ 60 independent saprotrophic lineages, many having evolved from brown and white rot ancestors [24, 25]. The identity of the lineage of many ECM fungi is still unknown, but phylogenetic analyses show that their evolution is associated with a shift in nutritional mode and the differential loss of OM decomposition genes [24, 26]. Evidence shows that some ECM taxa have the capacity to decay soil organic matter to mobilize soil organic N but that their ability to decay and access N depends on their evolutionary origin [27, 28]. Thus, a closer look at N uptake and OM decomposition gene frequencies of individual ECM species compared to those of fungi closely related to prospective ECM phylogenetic ancestors can potentially further our understanding of the ecology and evolution of ECM fungi [e.g., 24].

## Materials and Methods

### Fungal Functional Trait-Based Classification

We collected public genomic data from the US Department of Energy 1000 Fungal Genomes Project (i.e., MycoCosm) and created a database comprising a total of 983 genomes, representing nine fungal phyla or subphyla, 118 orders, 296 families, and 571 genera [12, 29]. We obtained the internal transcribed spacer (ITS) region for each species present in our genome list through NCBI taxonomy [30] and GenBank (Supplementary Table 1). We then used the ITS region to classify species into functional trait-based groups (i.e., trophic mode, functional guild, growth morphology, and rot type) using FUNGuild [9]. A total of 104 genomes could not be assigned to any group, leaving 879 genomes for further analysis. Of these, 217 species fell under two or more categories for trophic mode and 248 species under two or more categories for functional guild. For these, we manually curated the list and designated a single category to each species (e.g., “final trophic mode” in Supplementary Table 1). If a species’ trophic mode was classified as pathotroph-saprotroph but its functional guild was plant pathogen, we classified the final trophic mode as pathogen. Similarly, if a species was described as pathotroph-saprotroph or saprotroph-symbiotroph or pathotroph-saprotroph-symbiotroph and belonged to a rot type, we classified it as saprotroph (the latter was only the case for one genome, *Acremonium* spp.). At the functional guild level, if a species was classified as undefined saprotroph-wood saprotroph or some other combination of functional guilds and belonged to a type of rot, we classified it as wood saprotroph (e.g., *Xylaria hypoxylon*). Moreover, we examined the description for each genome as given in MycoCosm; if the description provided insight into a species’ function (e.g., pathogenic), we used that information to designate the final trophic mode. If a species was classified into all three possible trophic mode categories (i.e., pathotroph-saprotroph-symbiotroph), and we were unable to convincingly designate one final category using the approaches described above (for < 5% of species, or 44 of 983 genomes), we did a literature search using Google Scholar and designated the final trophic mode based on the top ten most highly cited papers. In general, trait-based saprotrophs are categorized as undefined (i.e., general) or wood saprotrophs, symbiotrophs as endophytes or ECM, and pathotrophs as plant or animal pathogens. We excluded genomes from underrepresented groups (i.e., groups with *n* < 10 genomes), such as arbuscular mycorrhizal fungi, dung saprotrophs, litter saprotrophs, leaf saprotrophs, etc. to avoid drawing conclusions from insufficient data. A complete list of all genomes is available in Supplementary Table 1.

### Gene Frequencies of Fungal Functional Trait-Based Groups

We determined, for each genome, the number of gene copies for N uptake and OM decomposition gene families (Table 1) because gene copy number modulates gene expression and can contribute to significant phenotypic variation [31]. We used ammonium and nitrate transporter gene families as indicators for uptake potential of inorganic N; amino acid permeases as indicators for potential of organic N uptake; lignin peroxidase for the potential to modify lignin, a recalcitrant organic C compound found in many plants; and cellobiohydrolase, β-Glucosidase, and lytic polysaccharide monooxygenase for potential to decay cellulose, the most abundant polymer on Earth [32]. We chose these gene families because they represent important traits relevant to ecosystem N and C cycling processes [13, 33–35]. By using gene families rather than individual genes, we were able to cover a broader set of genes, some of which are not yet fully characterized for many fungal species but, that by homology, we know are present in their genomes. We calculated gene frequency as the number of genes per 10,000 genes to standardize for genome size, following a similar approach used by Treseder and Lennon [13] and Siletti and collaborators [36]. We grouped gene families into N uptake and OM decomposition groups (Table 1). For each, we carried out a one-way analysis of variance (ANOVA) with functional group as an independent categorical variable and N uptake or OM decomposition gene frequency as the dependent variable. We did similar analyses for each one of the different gene families and determined significant pairwise comparisons with Tukey HSD post hoc tests using a 95% confidence interval among groups. In all cases, we determined significant differences at *P* ≤ 0.05. We used *R* for all tests [37].

**Table 1:** Gene family domains encoding for enzymes responsible for N uptake and organic matter (OM) decomposition [13]

Group	Trait	Enzyme	Gene family domain
N uptake	Uptake of inorganic N	Ammonium transporter	IPR001905
		Nitrate transporter	IPR004737
	Uptake of organic N	Amino acid permease	IPR004762
OM decomposition	Decomposition of cellulose	Cellobiohydrolase	IPR001722
		β-Glucosidase	IPR001360
		Lytic polysaccharide monooxygenase	IPR005103
	Decomposition of lignin	Lignin peroxidase	IPR001621

#### Example #1: Using Linked Gene-Trait Data to Examine the Response of Fungal Functional Trait-Based Groups to Long-Term Simulated Nitrogen Deposition

The CNAS at the Harvard Forest LTER, initiated in 1988 to examine the long-term consequences of high cumulative rates of N additions to forest ecosystems [38, 39], consists of three 30 × 30 m mega plots subdivided into thirty-six 5 × 5 subplots which are treated as experimental replicates. One mega plot is the control (N0; ambient N deposition is currently 7–9 kg N ha^−1^ yr^−1^ at this site [40]) and the other two mega plots receive one of the following treatments as liquid NH_4_NO_3_ fertilizer on a monthly basis during the growing season: N50 (50 kg N ha^−1^ yr^−1^) or N150 (150 kg N ha^−1^ yr^−1^). The N50 plot represents an N deposition scenario predicted for 2050 [41], while the N150 plot represents an N saturation scenario. Previous work has confirmed strong treatment effects not caused by random variation [16, 19, 23, 42, 43].

We used previously published soil fungal ribosomal RNA (rRNA) data collected from the CNAS [42] to investigate if knowing something about the frequencies of N uptake and OM decomposition genes of fungal functional groups provides insight into observed fungal responses under simulated N deposition. To answer this question, we calculated the N response as the total number of RNA reads in the treated relative to control plots (Supplementary Table 2). We used RNA because RNA is considered an indicator of “metabolically active” taxa [44]. We grouped reads by taxa, assigned functional categories using FUNGuild, and calculated N response as:

$$ Nitrogen\ Response=\left(\frac{N50\  or\ N150\  RNA\  reads}{N0\  RNA\  reads}\right)-1 $$

When RNA reads were present in control but absent in N50 or N150, we assigned a value of − 1 to indicate a decrease under N. When RNA reads were present in N50 or N150, but absent in N0, we assigned a value of 1 to indicate an increase under N. Any taxon that increased or decreased by a magnitude of 3 or more were eliminated from our analysis; the effect of these outliers was analyzed in Morrison and collaborators [42]. Despite removing outliers and working with total RNA reads rather than relative abundance data, our results were comparable to those reported previously [42]. We conducted a one-sample *t* test for each trait-based category and compared the mean N response to μ = 0. Trait-based categories with a significant positive N response had a greater abundance of RNA reads under N addition compared to control, while those with a significant negative N response had a lower abundance of RNA reads under N addition compared to control.

#### Example #2: Using Linked Gene-Trait Data to Gain Insight into the Life History Traits of Ectomycorrhizal Fungi

We extracted gene frequency data for all genomes listed as ECM fungi at the functional guild level (Supplementary Table 1). In addition, we calculated the average frequency for N uptake and OM decomposition genes for all genomes classified as brown rots, soft rots, and white rots. We asked, what can we learn about ECM life history traits by comparing the frequencies of N uptake and OM decomposition genes to those of fungi categorized by rot type (i.e., brown, soft, or white rot)? To address this question, we compared N uptake and OM decomposition gene frequencies of each ECM genome to the average gene frequencies for brown rots, soft rots, and white rots. A list of all ECM fungi, their gene frequencies, and their color-coded taxonomical classification is available in Supplementary Table 3.

## Results and Discussion

### Gene Frequencies of Fungal Functional Trait-Based Groups

**Fig. 1:**
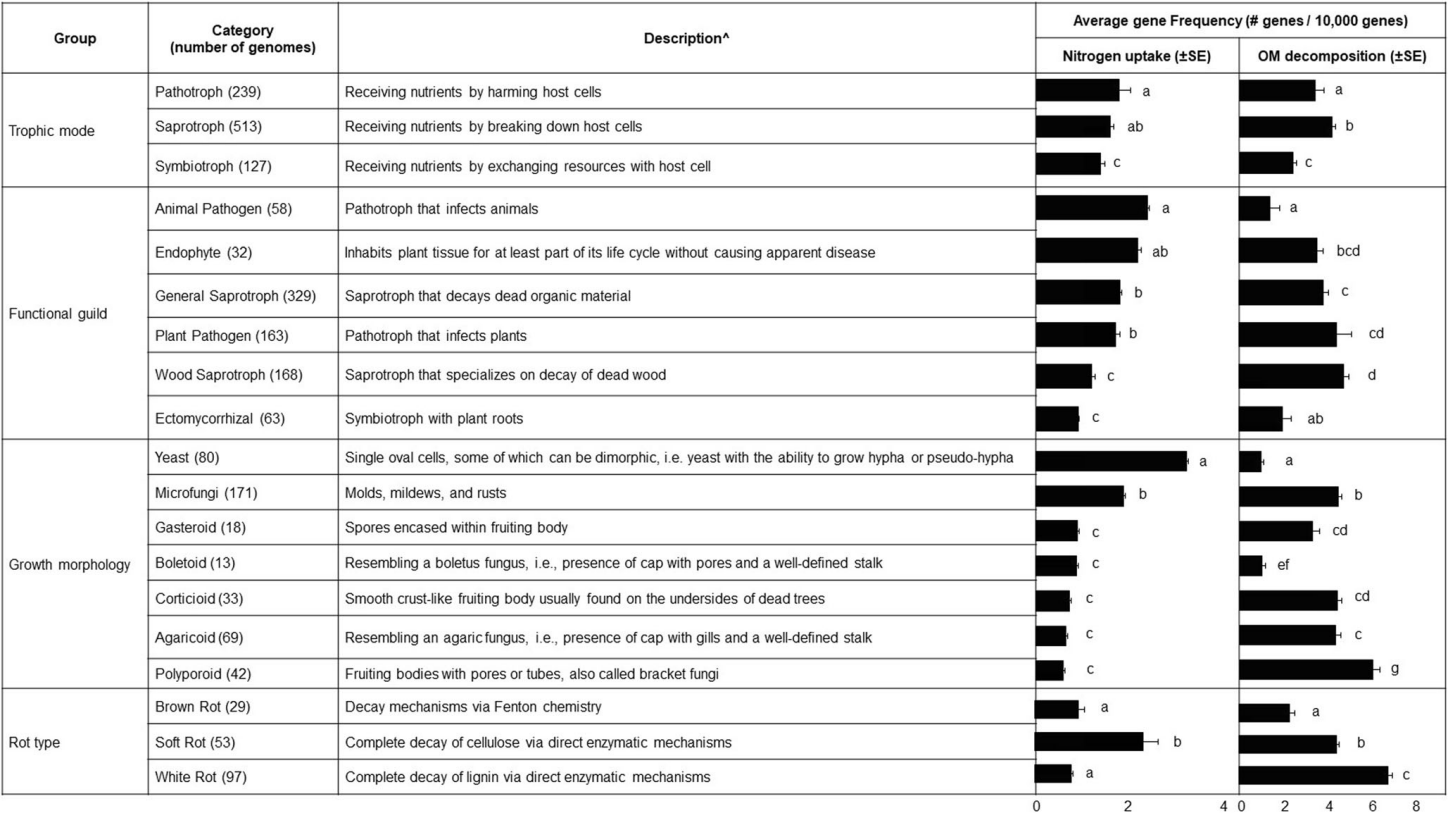
Frequency of N uptake and OM decomposition genes for each fungal trait-based category. Data represents the average gene frequency of analyzed genomes per category shown in parenthesis. Error bars indicate SE. Different compact letters denote post hoc significant differences (*P* ≤ 0.05) in average N uptake or OM decomposition gene frequency with category used as independent variable within each trait-based group. ^References: [9, 45]

The average frequency of N uptake and OM decomposition genes varies widely, both within and across fungal functional groups (Fig. 1). Even though the uptake of N and decomposition of OM are both energetically expensive processes, we did not find evidence at the gene level that fungi preferentially invest more heavily in one of these processes at the expense of the other, suggesting both are critical to the ecological success of fungi. That is, there was no significant negative correlation between N uptake and OM decomposition gene frequencies (Supplementary Fig. 1). There are a few notable exceptions to this general finding, namely, that yeasts and animal pathogens have high N uptake, but low OM decomposition gene frequency, while white rots and polyporoids display the opposite pattern.

**Fig. 2:**
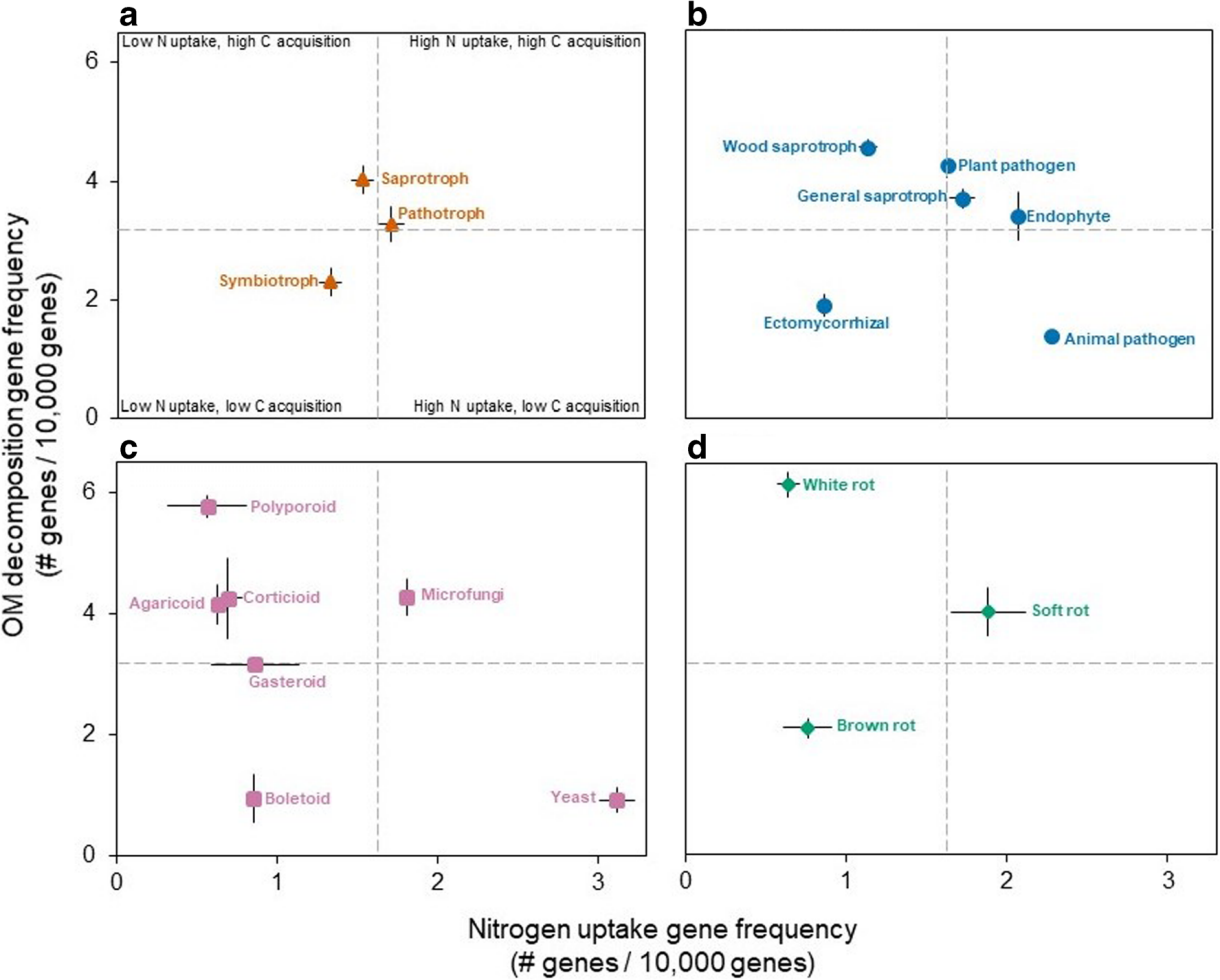
Trait space showing N uptake versus OM decomposition gene frequencies by trait-based group

Examining the trait space of N uptake versus OM decomposition gene frequencies for each trait-based group (Fig. 2), we see that trophic mode (Fig. 2 A) offers narrow resolution across categories, especially between saprotrophs and pathotrophs, compared to the other three categories which show broad resolution (Fig. 2, B, C, and D). When the trait space is divided into quartiles, we see that symbiotrophs, ECM fungi, boletoids, and brown rots have, in general, low N uptake and OM decomposition gene frequency. Contrastingly, general saprotroph, microfungi, and soft rots have a relatively high frequency of N uptake and OM decomposition genes. Wood saprotrophs, polyporoids, agaricoids, corticioids, and white rot fungi have low N uptake but high OM decomposition gene frequencies, while animal pathogens and yeasts have high N uptake, but low OM decomposition gene frequencies. Fungi with significantly higher frequency of N uptake genes (i.e., yeasts) are known for their ability to withstand stress and grow in a variety of N sources [46–49]. Contrastingly, fungi with significantly higher frequencies of OM decomposition genes (i.e., polyporoids, wood saprotrophs, and white rots) are adapted to survive in carbon-rich environments such as rotting wood, as well as to exploit complex carbon substrates, such as lignin [50, 51]. This type of broad trait-based information can be useful for ecological trait-based modelling, as generalities can more easily be turned into parameters to input in mathematical models [52, 53]. In addition, terrestrial biosphere models require generation of belowground trait-based ecological information, such as we provide here, to inform model inputs of fine spatial and temporal resolution models [54].

**Fig. 3:**
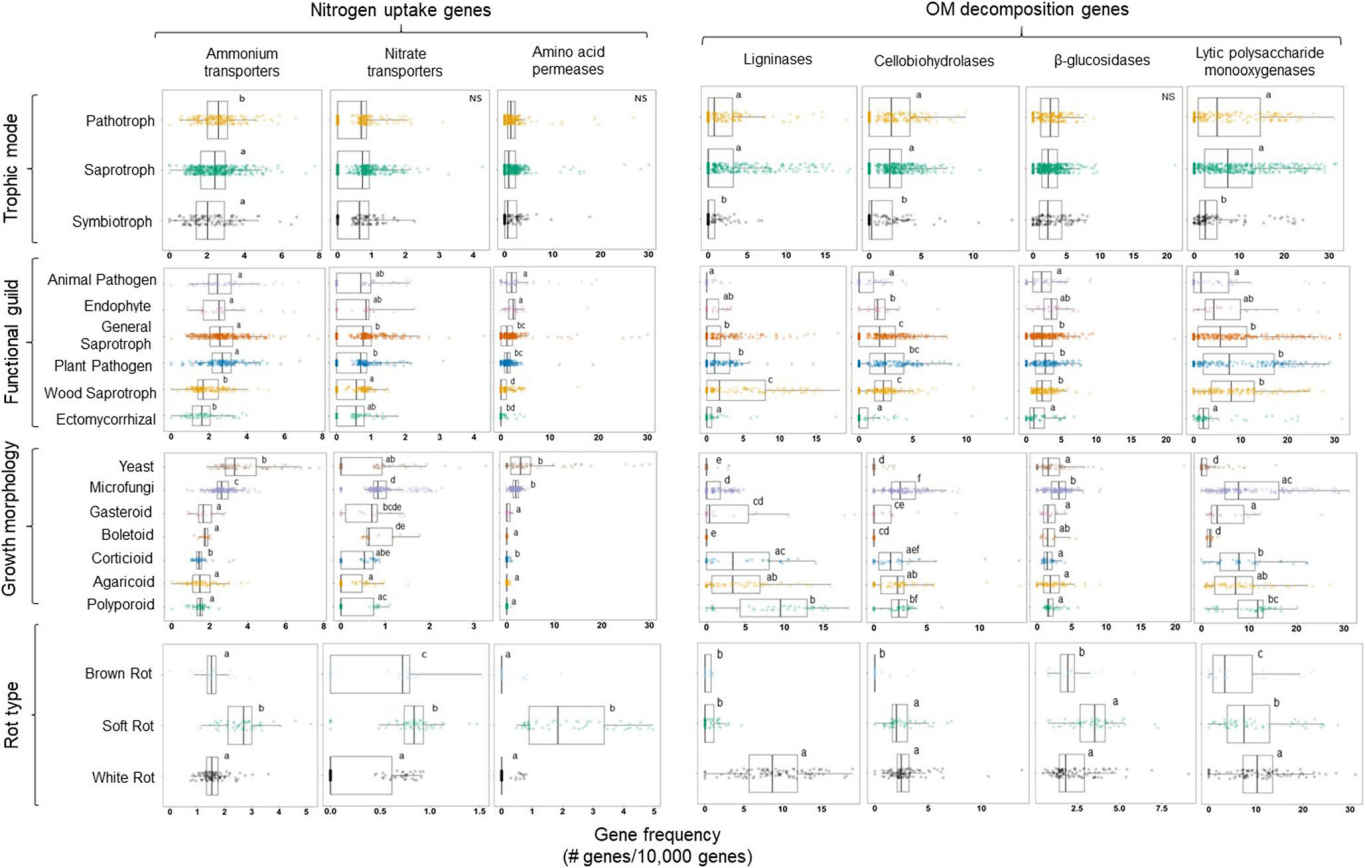
Box and whisker plots showing the distribution of gene frequencies with lower and upper quartiles, average, and lowest and highest observations plotted. Each point represents an individual genome. Different letters denote significant differences within each gene family and fungal trait-based group (*P* ≤ 0.05). Detailed information on each genome can be found in Supplementary Table 1

However, for each fungal group and gene family, results were nuanced (Fig. 3). Disaggregated data shows that there are outliers present in every trait-based group for every gene family. For example, even though the average ammonium transporter gene frequency is significantly different between the general saprotroph and ECM categories, genomes on the lower end of general saprotrophs overlap with the mean value of ECM. But as discussed by others [55], trait-based approaches are intended to move beyond “individual-centric” or “species-centric” results. This is especially true for trait-based approaches in ecosystem or global-scale ecology where a few outliers likely have no major impact on large-scale processes [56]. However, some challenge this view and argue that outliers can help us better understand ecosystem functioning and that the relative abundance of outliers needs to be considered [57].

#### Example #1: Response of Fungal Functional Trait-Based Groups to Long-Term Simulated Nitrogen Deposition

**Fig. 4:**
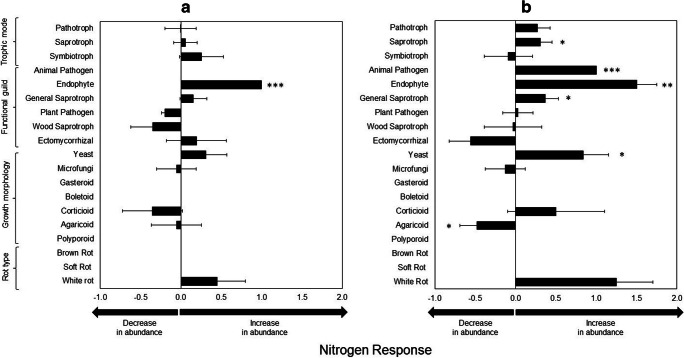
Nitrogen response of fungal functional trait-based groups based on total rRNA reads as measured by high-throughput sequencing of soil collected from (A) N50 and (B) N150 plots at the Chronic Nitrogen Amendment Study at the Harvard Forest LTER site. Bars show average ± SE. Results are significant when means are significantly higher or lower than a mean of 0 (*t* test *p* = 0.05 (*), *p* ≤ 0.01 (**), *p* ≤ 0.001 (***). A significant positive response represents an increase in abundance of rRNA reads under elevated nitrogen, while a significant negative response indicates a decrease

Over three decades of research at the CNAS at the Harvard Forest LTER, it has shown that long-term simulated N deposition results in OM accumulation [16–18] partially due to shifts in the fungal community [23]. Our trait-based analysis of Morrison and collaborators’ data [23] showed that saprotroph, animal pathogen, endophyte, general saprotroph, and yeast categories have a positive N response at the highest N treatment (i.e., N150) (Fig. 4, Supplementary Table 2). In contrast, agaricoids showed a significant negative N response. Endophytes were the only category with a significant N response at both levels of N addition. Plant pathogen, wood saprotroph, and ectomycorrhizal fungi exhibited negative N responses at N50 or N150, but these were not significant. Similarly, white rot fungi showed a positive, but non-significant N response.

All the trait-based categories showing a significant positive N response had high N uptake gene frequency (Fig. 1). Specifically, animal pathogen, endophytes, general saprotroph, and yeast have high gene frequency for ammonium transporters and amino acid permeases (Fig. 3). Trait-based categories with higher gene frequency of ammonium transporters, such as yeasts, might be better at metabolizing N, which is very abundant at the CNAS and may provide them with a competitive advantage over groups with lower gene frequency. For example, the yeast *Yarrowia* spp. can use many different organic and inorganic N sources [58, 59]. The relatively higher frequency of ammonium transporter genes is likely the reason underlying this ability; moreover, transmembrane N transporter genes in yeasts are prone to selection and are rapidly evolving [60–62]. It is important to note that even though soft rots have a high N uptake gene frequency, we did not include them in our analyses because they were not sufficiently replicated in our Example #1 dataset and did not allow for proper statistical analyses. However, two soft rot taxa had a positive N response at N50, and three taxa had a positive N response at N150 (Supplementary Table 2). Nonetheless, gene frequency does not necessarily indicate higher gene expression but does suggest functional potential. As the field of microbial trait-based ecology develops, linking gene expression with gene frequency and traits will be essential.

Our first example suggests that using linked gene-trait data is predictive of how the fungal community will respond under N deposition, but only when the system is exposed to extreme conditions (i.e., high levels of exogenous N) as most N responses were significant at N150 (Fig. 4B). Regardless, using trait-based groupings provides more predictive power than taxonomy; when analyzed at the order level, taxonomic classification provided no clear predictive power (Supplementary Fig. 2). For example, the Hypocreales and Chaetothyriales displayed a significant positive N response (Supplementary Fig. 2A) but neither one of those taxonomical orders stands out for having high N uptake gene frequency (Supplementary Fig. 2B). In addition, the trait-based categories included in our study are scattered across the entire fungal tree of life (Supplementary Fig. 3); thus, phylogenetic approaches for predicting fungal traits which are relevant at the ecosystem scale may be challenging. Even though other studies have shown that certain fungal traits are phylogenetically conserved across taxa [e.g., 63], some of these traits may not be relevant for ecosystem-scale functional processes [64]. But phylogenetic analyses of ecologically relevant traits could provide a better understanding of the evolution and life history traits of fungi. This is particularly important because others have found phylogenetic signals for certain OM decomposition enzymes where decomposition abilities were more similar among phylogenetic lineages than among functional guilds [65]. Most trait-based categories in FUNGuild, as well as the gene families we included in our analysis, may be too broad to identify phylogenetic signals, whereas examining specific traits, such as the ability to produce specific enzymes encoded by a single gene, may be a better approach for searching for phylogenetic signals.

#### Example #2: Insight into the Life History Traits of Ectomycorrhizal Fungi

**Fig. 5:**
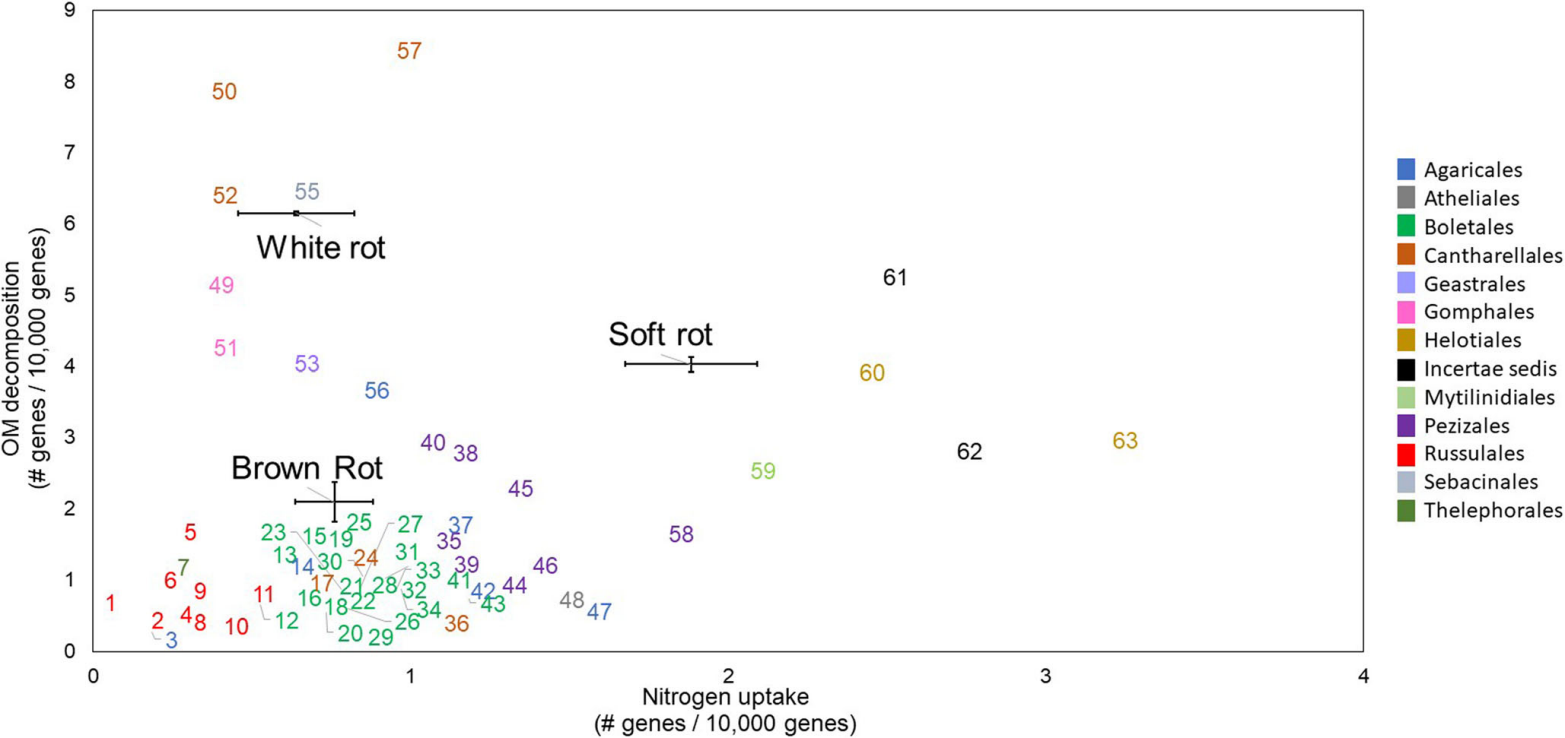
Frequency of nitrogen uptake and OM decomposition genes for individual ectomycorrhizal taxa (numbered) in contrast with the average gene frequency for white, brown, and soft rot fungi (labeled in graph). Identification number, species, and other taxonomical information are provided in Supplementary Table 3

The evolutionary histories of ECM fungi are challenging to study because of the polyphyletic nature of the ECM niche and the diversity of saprotrophic lineages from which ECM fungi evolved [24, 25]. A comparison of the frequencies of N uptake and OM decomposition genes of individual ECM fungi with those of different fungal rot types shows most ECM fungi with sequenced genomes (at least those available in MycoCosm) have gene frequencies similar to brown rot fungi (Fig. 5, Supplementary Table 3). However, the clustering of ECM genomes with the genomes of brown rot fungi likely reflects at least two evolutionary trajectories: a shared evolutionary history, for example, various Boletales, appears to have derived from brown rot lineages [24] and in these cases clustering probably reflects common ancestry. But various other genomes with gene frequencies similar to brown rots, including species of the Russulales and Pezizales, are not phylogenetically close to brown rot clades [66]. These genomes highlight the potential for a second evolutionary path involving convergent evolution towards a particular gene space. Both trajectories result in similar frequencies of N uptake and OM decomposition genes.

On the whole, sequenced ECM genomes appear to have divergent functional potentials for both OM depolymerization and N uptake. Genomes with similar gene frequency distributions to white rot fungi, for example, the Cantharellales and Gomphales, are likely to have vastly different effects on ecosystem C and nutrient cycling compared to ECM more similar to soft rot fungi, for example, the Helotiales. A recently published comprehensive study of 62 mycorrhizal species also suggests a broad variation in the capacity of mycorrhizal fungi to acquire C from soil OM [67]. Nonetheless, most ECM genomes appear in brown rot gene space (Fig. 5): whether the pattern reflects a real ecological dynamic or is an artifact of the genomes available to date is unknown, but we suggest it would be useful for future sequencing efforts to target organisms appearing to fall in between the different rot types (Fig. 5), potentially by including species of the Geastrales, Agaricales, and Mytilinidiales. We targeted specific sets of genes in our analyses (Table 1) but at least a few ECM fungi take alternate routes to depolymerize carbon (e.g., Fenton chemistry) [68–70]. Additional analyses targeting different sets of genes or measuring enzyme production may reveal additional capacities for OM decomposition or provide additional information about patterns in gene space (Fig. 5).

## Conclusion

The variety of traits in fungi is vast [8, 14, 53, 71]. In this study, we examined trait-based groups as defined by FUNGuild and focused on seven broad gene families, with this dataset being useful for addressing specific questions that concern N uptake and OM decomposition. We found that the average frequency of N uptake and OM decomposition genes varies across fungal trait-based groups and within trait-based categories. The N uptake–OM decomposition trait space across different functional groups showed differential resolution. We show that using linked gene-trait data can provide a better understanding and potentially predict how fungi will likely respond to environmental stress under extreme conditions. These predictions are especially relevant, given that these conditions are becoming more frequent due to global climate change. Moreover, we show that exploring linked gene-trait data can help us to understand life history traits in fungi. Our work provides a framework to explore N uptake and OM decomposition gene frequencies in fungal trait-based groups and address ecological questions from a mechanistic view.

Since many ecological modelling trait-based efforts often focus on tradeoffs [72–74], it is essential to mention that we did not find evidence of tradeoffs at the gene frequency level (i.e., higher N uptake and low OM decomposition gene frequency or vice versa). However, this does not suggest that gene frequency level tradeoffs for other important traits and processes are not present in trait-based groups. For example, exploring gene frequencies of stress tolerance genes may reveal a gene-level tradeoff when compared to OM decomposition [72]. Furthermore, higher gene frequency might not directly relate to gene expression; thus, our results only suggest the potential to take up different forms of N and to decompose different OM compounds. Gene expression data on trait-based groups is still needed. Regardless, our results are some of the very first to link gene frequencies to fungal traits and showcase the use of linked gene-trait data in two specific examples.

## Supplementary Information

ESM 1 (DOCX 1.01 mb)

ESM 2 (XLSX 369 kb)

ESM 3 (XLSX 22.6 kb)

ESM 4 (XLSX 18.8 kb)

## Data Availability

All data used in this manuscript are included in the main text or as supplementary information.
