# Supplementary material for: Linking Genes to Traits in Fungi
Source: Microb Ecol. 2021 Jan 22;82(1):145–55. doi: 10.1007/s00248-021-01687-x (PMC8282587; doi:10.1007/s00248-021-01687-x)

**Supplementary Figure 1.** Linear regression between N uptake and OM decomposition gene frequency of each genome present in our dataset (Supplementary Table 1). Red line is best fit.

**
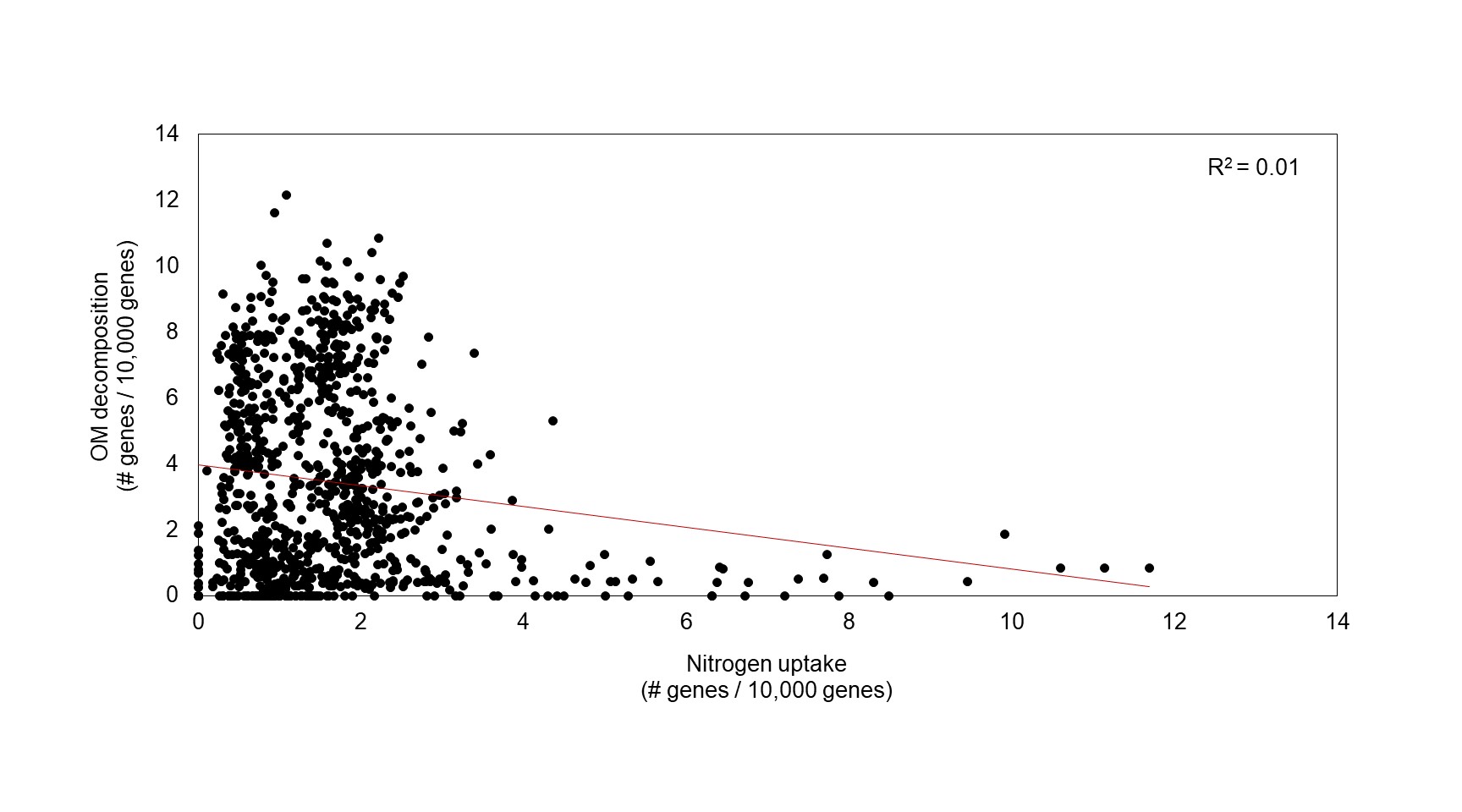
**

**Supplementary Figure 2.** A) N responses of fungal orders in the CNAS at the Harvard Forest LTER. Bars show average ± SE. Results are significant when means are significantly higher or lower than a mean of 0 (*t*-test p = 0.05 (*), p ≤ 0.01 (**), p ≤ 0.001 (***). A significant positive response represents an increase in abundance of rRNA reads under elevated nitrogen, while a significant negative response indicates a decrease. B) Gene frequencies of N uptake and OM decomposition of most taxonomical orders included in our database (Supplementary Table 1).

1.
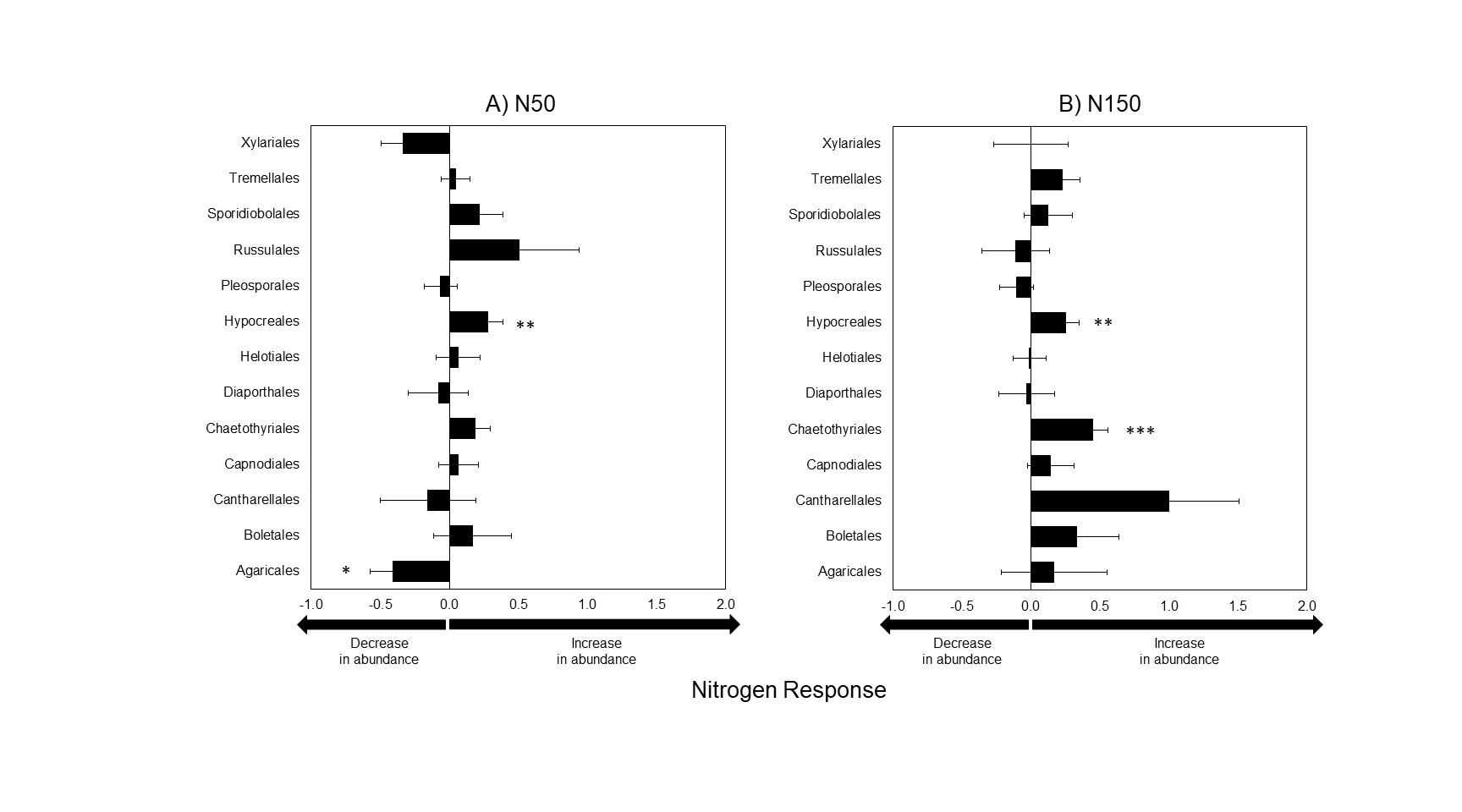


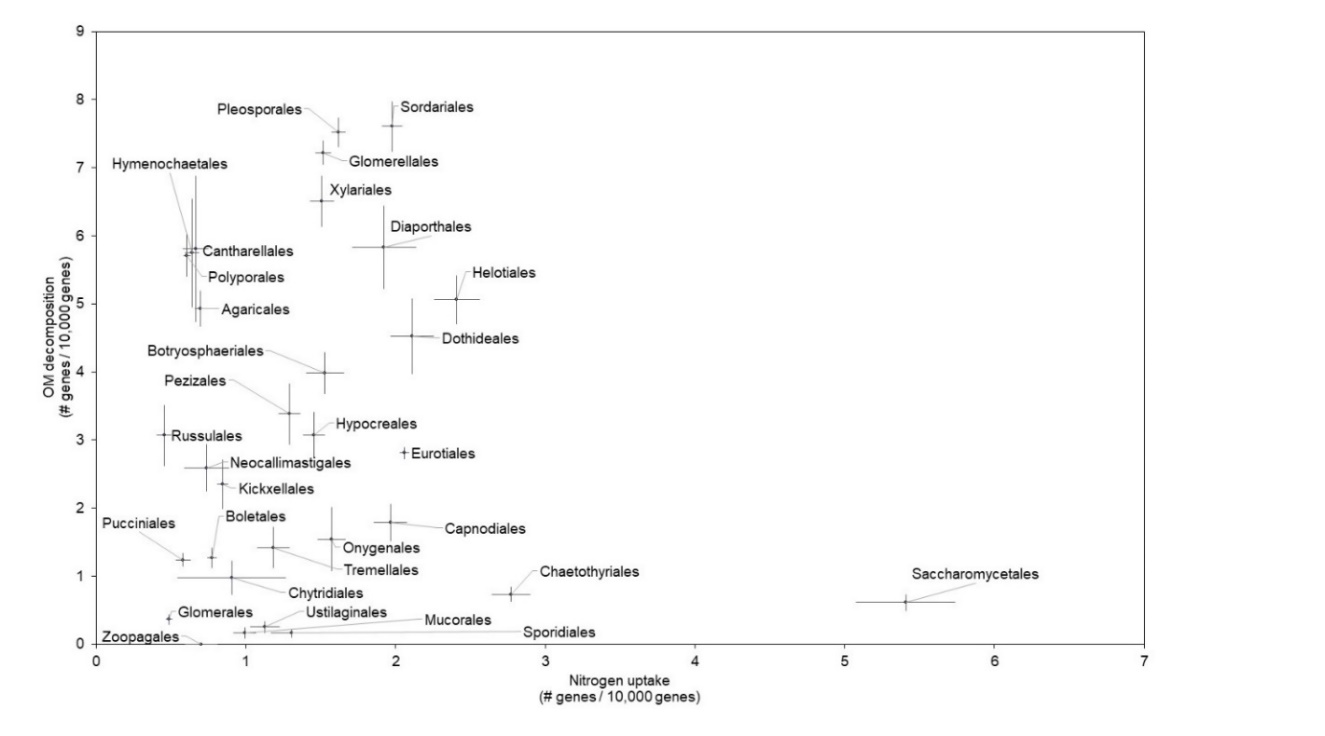


**Supplementary Figure 3.** Taxonomic distribution of fungal trait-based groups in A) pathotrophs; B) saprotrophs, where the left-side panel shows the Basidiomycota, the center panel shows the Ascomycota, and right-side panel the non-Dikarya (i.e., non-Ascomycota and non-Basidiomycota) fungi; and C) symbiotrophs.

1. Pathotrophs


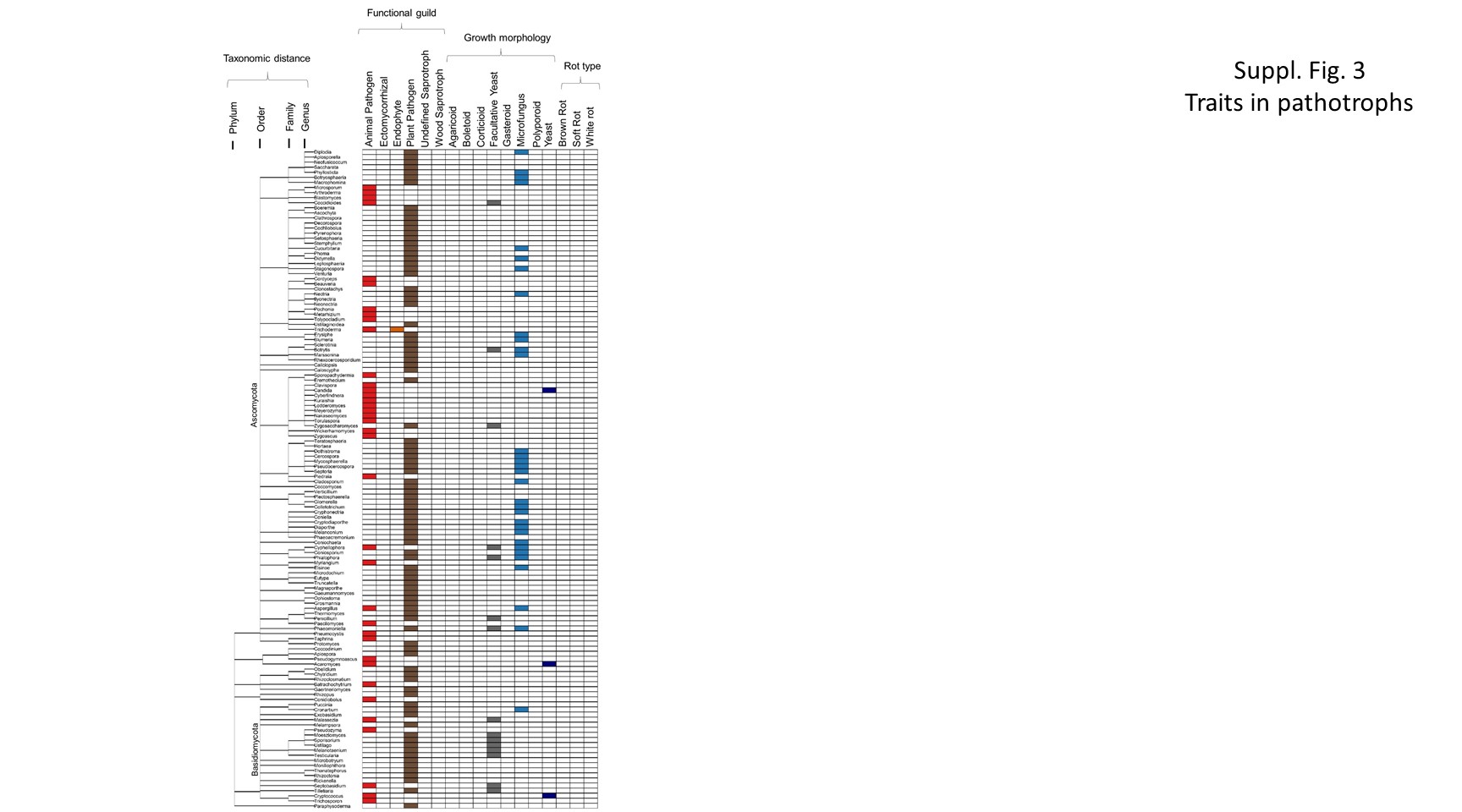


1. Saprotrophs


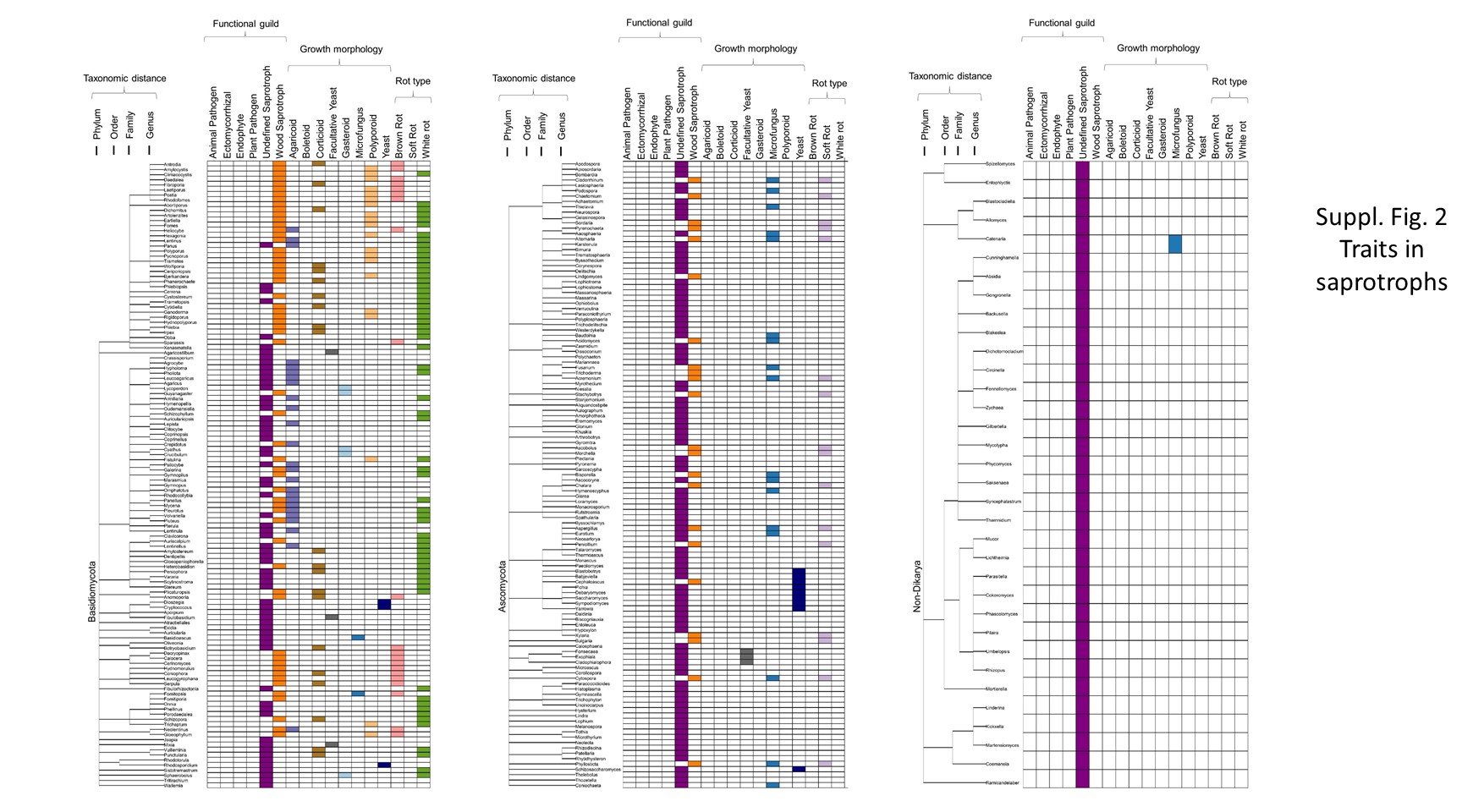


1. Symbiotrophs


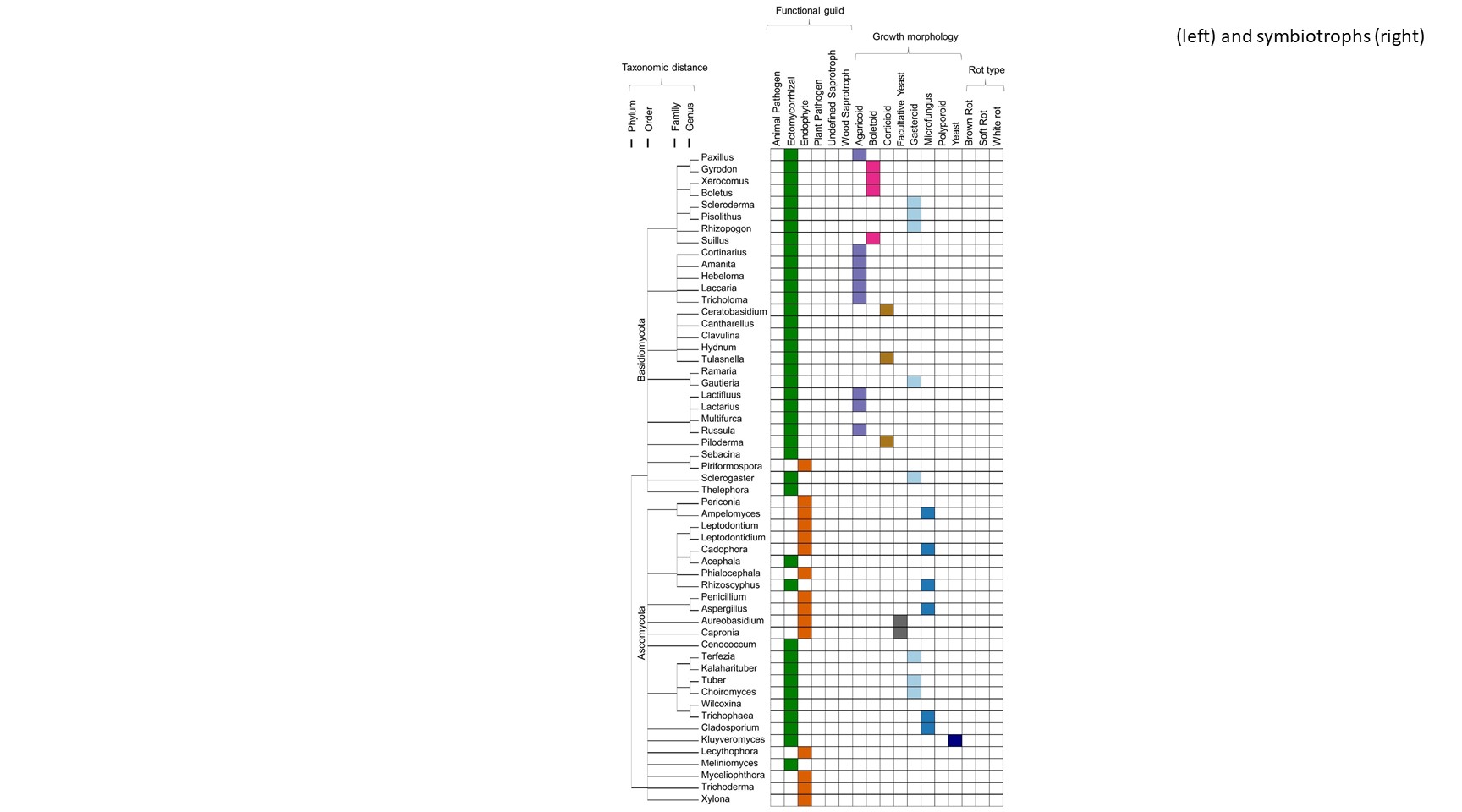

Supplement: Supplementary file 1 — (DOCX 1.01 mb) [file 248_2021_1687_MOESM1_ESM.docx]
